# Characterization of *Streptomyces* spp. Isolated from the Sea Surface Microlayer in the Trondheim Fjord, Norway

**DOI:** 10.3390/md6040620

**Published:** 2008-12-01

**Authors:** Sigrid Hakvåg, Espen Fjærvik, Kjell D. Josefsen, Elena Ian, Trond E. Ellingsen, Sergey B. Zotchev

**Affiliations:** 1 Department of Biotechnology, Norwegian University of Science and Technology, N-7491 Trondheim; 2 SINTEF Industrial Biotechnology, SINTEF, N-7034 Trondheim, Norway

**Keywords:** Sea surface microlayer, streptomycetes, antimicrobial activity, phylogenetic analysis

## Abstract

The water surface microlayer is still poorly explored, although it has been shown to contain a high density of metabolically active bacteria, often called bacterioneuston. Actinomycetes from the surface microlayer in the Trondheim fjord, Norway, have been isolated and characterized. A total of 217 isolates from two separate samples morphologically resembling the genus *Streptomyces* have been further investigated in this study. Antimicrobial assays showed that about 80% of the isolates exhibited antagonistic activity against non-filamentous fungus, Gram-negative, and Gram-positive bacteria. Based on the macroscopic analyses and inhibition patterns from the antimicrobial assays, the sub-grouping of isolates was performed. Partial 16S rDNAs from the candidates from each subgroup were sequenced and phylogenetic analysis performed. 7 isolates with identical 16S rDNA sequences were further studied for the presence of PKS type I genes. Sequencing and phylogenetic analysis of the PKS gene fragments revealed that horizontal gene transfer between closely related species might have taken place. Identification of unique PKS genes in these isolates implies that de-replication can not be performed based solely on the 16S rDNA sequences. The results obtained in this study suggest that streptomycetes from the neuston population may be an interesting source for discovery of new antimicrobial agents.

## 1. Introduction

Search for new biologically active microbial secondary metabolites is important in order to meet the increasing demand for new antibiotics. Actinomycetes, especially those belonging to the genus *Streptomyces*, are known to produce a wide variety of biologically active compounds. *Streptomyces* bacteria were reported to produce ~70% of the currently characterized actinomycete natural products [1]. However, most of the *Streptomyces* characterized up to date were isolated from the terrestrial environment, while those originating from marine sources still remain poorly explored.

In an environment with high densities of metabolically active bacteria, competition is likely to be fierce, and properties such as production of antibiotics may give organisms an advantage. A number of antibiotic producers have been isolated from the marine environments [2, 3], and experimental data indicate that production of antibiotics could play an important role in the competitive relationship within the marine bacterial populations [4]. Antagonistic interactions among soil-living microorganisms are well documented, and are attributed to the production of antibiotics by certain bacteria and fungi in environments rich in organic material [5, 6]. Recently, the same trend has been discovered for marine microorganisms, which are abundant in mesotrophic and eutrophic waters or during phytoplankton blooms [7].

In a study of antagonistic interactions among marine pelagic bacteria it was found that more than half of the isolates expressed antagonistic activity, and this trait was more common among particle-associated (66%) than free-living bacteria (40%) [8]. Particles often tend to accumulate at the sea surface, and the aquatic surface layer contains a series of sub layers [9]. Neuston is a collective name for the life forms in the surface layer of oceans and lakes, and can be divided into epineuston and hyponeuston. Epineuston organisms live on the top of the water surface, and are naturally dependent on the surface tension of the water. Hyponeuston organisms live in the top few centimetres of the water column. High densities of metabolically active bacteria, often called bacterioneuston, are found in the surface microlayer [10-13].

Norwegian marine ecosystems have developed in a rather cold and severe climate, suggesting that the selective pressure on microorganisms comprising parts of such systems must have been quite unique (cold seawater environment). Because of this, it seems likely that these microorganisms have developed antibiotic biosynthesis pathways that differ from those utilized by terrestrial microorganisms. Even though the diversity of microorganisms in the marine environment is high, only a minor fraction (less than 1 %) can be cultivated in the laboratory, presumably because of failure to mimic the natural growth conditions [14]. In this work we isolated *Streptomyces* bacteria from the surface microlayer in the Trondheim fjord (Norway). The isolates were characterized using molecular taxonomy, assays for antimicrobial activity and presence of polyketide synthase genes.

## 2. Results and Discussion

### 2.1. A large proportion of cultivable neuston actinomycetes produce antimicrobial compounds

Bacteria morphologically similar to streptomycetes were isolated from surface microlayer collected at Steinvikholmen (a small islet) and in the Åsen fjord in the Trondheim fjord, Norway. The water temperatures during sampling were 4.3 and 5.8 °C, respectively, and the air temperature was 3 °C in both cases. Water was sampled from two sites, both to increase the number of isolates and to possibly detect any spatial variations in diversity. Collecting water samples close to the shore increases the risk of cultivating terrestrial bacteria that have been washed into the sea. Initial isolation of the bacteria was therefore performed on agar media with 50 % seawater to increase the chance of isolating bacteria adapted to the marine environment.

Total numbers of bacteria isolated on Actinomycete isolation seawater agar with cycloheximide and nalidixic acid added to inhibit the growth of fungi and Gram-negative bacteria, were 2.5x10^3^ and 1.2x10^4^ cells/ml seawater from the two sites, respectively. Presumed actinomycetes (based on colony morphology) accounted for 9.8x10^2^ and 1.3x10^3^ cells/ml, respectively. From these, a total of 217 colonies from samples 1 and 2 represented by 134 and 83 isolates, respectively, were selected for further analyses.

Previously, it has been reported that bacteria isolated from the surface microlayer at coastal stations in the north-western Mediterranean Sea, contained an average of Gram-positive cultivable bacteria ranging from 2.3x10^3^ (France) to 3.0x10^4^ (Spain) ml^−1^ [15], indicating that the cell number can vary considerably depending on the sampling site. Based on these reports, the total number of isolates in the samples collected in this study is assumed to reflect at least some of the diversity in the Trondheim fjord.

Based on the colony morphology (colour of substrate and aerial mycelia, pigment production), the isolates could be divided into 10 groups, shown in Table 1.

**Figure 1.**
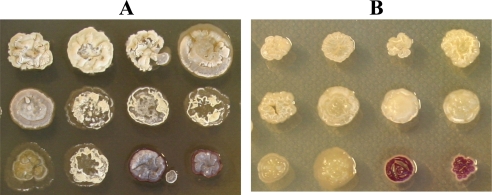
Growth of isolated actinomycetes after 7 days of incubation at 30 °C on ½ ISP2 agar with (A) and without (B) 50% seawater.

Cultivation of the isolates on agar medium with and without seawater, showed that they all grew better/faster on media with 50 % seawater added, as exemplified on Figure 1. Actinomycetes isolated from marine sediments have earlier been analyzed for their seawater requirement for growth [16]. The detected requirement has been interpreted as indication of marine origin or marine adaptation. None of the isolates in this study demonstrated inhibited growth on salt-containing media, suggesting that all isolates are marine bacteria or terrestrial bacteria adapted to the marine environment, and presumably occur naturally in the surface microlayer.

In order to explore the potential of the isolates to produce antimicrobial compounds, extracts from the colonies grown on three different solid agar media were tested in microbial inhibition assays. After an appropriate incubation time (depending on the growth rate), the plates with cells were dried, and extracted with DMSO. The extracts were tested in agar diffusion assays for antimicrobial activity. The initial assays were performed with *Micrococcus luteus* ATCC 9341*, Candida albicans* ATCC 10231 and *Escherichia coli* K12 as indicator organisms. The antimicrobial activity, presented in Table 2, is the total combined activity displayed by the isolates when grown on any of the 3 agar media. As expected, a high share of the isolates exhibited antimicrobial activity. In particular, 79% of the sample 1 isolates, and 85% of the sample 2 isolates showed antagonistic activity against at least one of the indicator organisms. Several of the isolates showing antimicrobial activity were active against more than one indicator organism, as shown in Table 2. This was particularly evident for the isolates with antibacterial activity, where around 80% of the isolates inhibiting Gram-negative bacteria also inhibited Gram-positive bacteria, and vice versa. Table 2 shows how the different inhibition patterns are distributed among the isolates from different morphological groups. The fact that some isolates displayed activity against more than one indicator organism may indicate production of several antimicrobial compounds and/or production of compounds with multiple microbial targets.

The percentage of neuston actinomycete isolates displaying antimicrobial activity was found to be considerably higher than those reported previously. In the earlier studies, about 50% of isolated marine pelagic bacteria exhibited antagonistic properties against other pelagic bacteria [8], and only 44 % of streptomycetes from the marine sediments have shown antibacterial activity [17]. In the latter study, 17% of the isolates displayed antifungal activity. A noticeably lower degree of antifungal compared to antibacterial activity among *Streptomyces* species isolated from marine sediments has also been reported by [18]. In our study, about 40% of the assumed (based on morphology and inhibition patterns) non-identical isolates from both sample 1 and 2 showed antifungal activities.

The methods chosen for surface sampling and cultivation may facilitate isolation of some types of bacteria over others. This will affect both the quantity and the diversity of the samples. Isolates from group G1 were frequently found in both samples. This may be due both to the fact that the selected growth conditions were best suited for the G1 isolates, and that they were in fact abundant in the surface microlayer. The diversities of streptomycete-like bacteria within the samples 1 and 2, at least as judged from colony morphology and inhibition patterns, were quite similar. This is probably not surprising, considering that the currents in the fjord continuously mix the water, thereby homogenizing the content of bacterioneuston to some extent.

Groups G5 and G6 had the highest share of isolates without any detectable antimicrobial activity under the conditions used. About half of these isolates displayed neither antifungal nor antibacterial activity. In both groups, one third of the isolates showed antifungal activity. Similarity in inhibition patterns was also noticeable between the G8 and G9 isolates. In these two groups, all isolates exhibited antimicrobial activity, whereof two thirds showed activity against both *M. luteus* and *E. coli*, and the rest also had activity against *C. albicans*. In addition G3 and G4 isolates showed a high degree of antibacterial activity, 77 % and 100 %, respectively. In total these results display a weak connection between morphology and antimicrobial activity to some extent.

Analysis of the 16S rDNA from the isolated bacteria reveals discrepancy between phenotypic grouping and the molecular taxonomy.

In order to reveal the diversity among isolated actinomycetes, a limited analysis of 1351 nt 16S rDNA gene fragments was performed. In total, 16S rDNA fragments from 46 isolates representing different groups distinguishable by morphology and inhibition patterns were amplified and sequenced. Alignment of the sequences showed a relatively high degree of homology within the candidate collection, suggesting replication of some isolates. However, several of the isolates representing potential replicates based on the 16S rDNA sequence, displayed unique inhibition patterns, indicating that they are not identical.

BLAST searches for the obtained sequences showed that the 16S rDNAs from all except 4 isolates had at least 99 % identity to sequences from *Streptomyces* spp isolated from marine sediments and sponges [19-22]. A widespread distribution of these bacteria in marine environments is consistent with the fact that they thrive on the salt-containing media.

**Figure 2.**
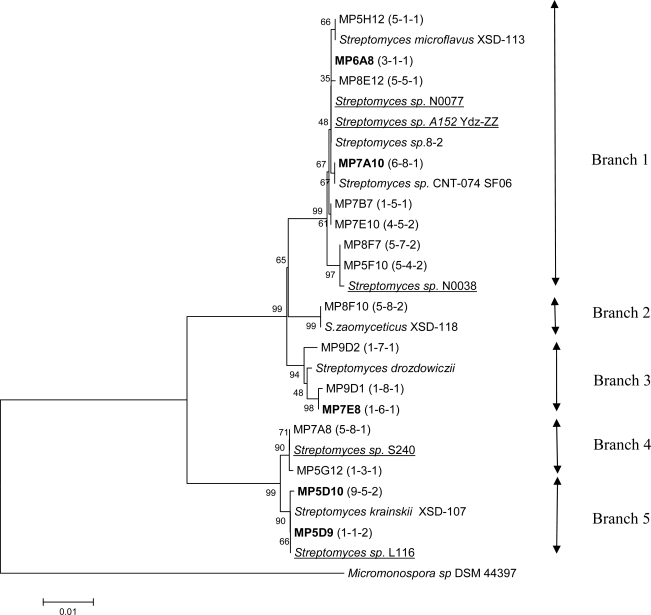
Phylogenetic tree constructed for partial 16S rDNA sequences (1351 bp) of 46 streptomycetes isolated from the surface microlayer in the Trondheim fjord, Norway. The tree also contains some of the closest matches from BLAST searches. The 16S rDNA sequence from *Micromonospora sp* DSM 44397 is included to root the tree. Numbers in brackets (x-y-z) refers to x: morphology group, y: inhibition pattern (see table 2), and z: sample number. Arrows indicate the different branches of the tree. Bold font indicates sequences representing several isolates. Strains of marine origin are underlined.

A phylogenetic analysis of the partial 16S rDNA sequences, displayed in Figure 2, was performed to reveal the taxonomic relationship between the different subgroups. In cases where several isolates had identical sequences, only one sequence was included in the analysis, without regard to differences in the morphology and inhibition patterns. As noted above, at least 10 morphologically different groups could be distinguished among the isolates. No clustering of these groups was observed in the phylogenetic analysis. Only minor grouping of isolates sharing the same inhibition patterns could be found. However, some clustering of isolates displaying either antifungal or antibacterial activity could be identified. In several cases, the “closest match” strains were reported to have antimicrobial activity that may be interesting from a commercial point of view.

Sequence for the isolate MP7A10 represents a group of six isolates, whereof five appear to have the same morphology (group G6). These isolates display different inhibition patterns, strongly suggesting that 16S rDNA gene sequences alone can not be used for dereplication of isolates.

Sequence for the isolate MP6A8 represents ten isolates with varying morphology (group G1-G5 and G10), of which eight were shown to have antibacterial activity. Including the remaining isolates in branch 1, a total of 17 out of 23 isolates in this branch displayed antibacterial activity, of which 15 were active against Gram–positive, and 2 against Gram-negative bacteria.

Three out of four isolates in branch 3 displayed antibacterial activity. The deviating isolate did not show any antimicrobial activity under the conditions tested. Sequence for isolate MP7E8 represents an additional isolate with the same inhibition pattern. Branch 4 and 5 consisted of isolates displaying both varying morphology and inhibition patterns. Isolate MP5D9 represents a group of 13 isolates whereof 8 displayed antifungal activity. There was a considerable variation in morphology among these isolates.

Several of the isolates with antibacterial activity (MP8F7, MP7E10, MP7B7, MP7E8 and MP9C8) showed 99 and 100% identity to *Streptomyces* species isolated from coastal sediments [19], but no antimicrobial activity has been reported for these species.

The sequence from isolate MP5F10 having antifungal activity showed 99% identity to *Streptomyces olivoviridis* and *Streptomyces sp.* N0028. The cultural appearance of *S. olivoviridis* agrees with that of MP5F10. This strain has been shown to produce a new antitumor antibiotic, thioviridamide, stimulating apoptosis signalling [23].

16S rDNA from the isolate MP9D2, which inhibits growth of Gram-positive bacteria, was 100% identical to that of *S. drozdowiczii* and *Streptomyces sp*. WL-2 (1351 bases). *S. drozdowiczii* NRRL B-24297 was reported to have cellulolytic activity [24], while *Streptomyces sp.* WL-2 produces xylanase [25].

16S rDNA from the isolate MP8F10 was 99% identical to *S. zaomyceticus* XSD-118. Different *S. zaomyceticus* strains have been shown to produce foroxymithine, narbomycin, picromycin and methymycin. Foroxymithine is an inhibitor of angiotensin-converting enzyme produced by actinomycetes, and may be of interest for medical use [26]. 16S rDNA from the isolate MP5H12 shows 99 % similarity to *S. microflavus* 173958.

### 2.3. Analysis of PKS gene fragments from selected streptomycetes isolates suggests horizontal gene transfer between closely related species

Polyketide synthases (PKSs) and/or non-ribosomal peptide synthetases (NRPSs) or a combination of these, are involved in production of many antimicrobial secondary metabolites in *Streptomycetes* and other bacteria, fungi, and plants. Screening for and analysis of PKS-I, PKS-II and NRPS genes in marine metagenomic libraries as well as soil samples have earlier been reported [27-30]. These analyses have been performed both to elucidate diversity and to pre-screen soil samples for identifying the ones most likely to contain producers of novel bioactive molecules.

Based solely on the 16S rDNA sequence analysis of our isolates, several of them seemed to be very closely related (i.e. had 100 % identical 16S rDNA fragments). At the same time, they showed different inhibition patterns in addition to displaying different morphology (Table 3). This fact prompted us to investigate the presence of PKS type I genes in a selected group of such isolates, which were chosen without considering morphology. In the phylogenetic analysis of 16S rDNA sequences (Figure 2), these isolates are represented by the isolate MP6A8 in branch 1.

Bacterial type I (modular) PKS gene fragments were amplified with the degenerate primers KSMA-F and KSMB-R [31], which can be used to amplify β-ketoacyl synthase (KS) domain encoding fragments of ca 700 bp. PCR with these primers resulted in amplification of fragments of expected size from all isolates, indicating their potential for production of polyketide secondary metabolites. Since PKS type I genes encode modular enzymes, and actinomycete strains usually contain more than one PKS gene cluster [28], it was expected that PCR products obtained with the KS-specific primers would represent mixtures of the KS-coding sequences. Therefore, sequencing of these gene fragments would be required for a better understanding of the diversity within the selected group of isolates and their dereplication.

PKS type I PCR fragments were cloned in *Escherichia coli* vector, and for each isolate 12 clones were sequenced. A total of 13 different sequences were obtained from 7 selected isolates. Six different KS-encoding sequences were amplified from the total DNA of the isolate MP8E7. BLAST search of the corresponding amino acid sequences revealed that fragments PKSI-1 and PKSI-6 from this isolate encode 95% identical KS domains showing 83% identity to the KS domain of MerC, a PKS involved in biosynthesis of the neuroprotectant meridamycin in *S. violaceusniger* [32]. The amino acid sequences for PKSI-2 and -3 fragments displayed 72% and 71% identity, respectively, to the PKS from *Saccharopolyspora erythraea* (unknown product) [33] and PteA2 PKS responsible for biosynthesis of antifungal polyene macrolide filipin in *S. avermitilis* [34]. Amino acid sequences of the PKSI-1, 2, 3 and -6 products were 68% to 95% identical, and might have been amplified from the same PKS gene cluster. The PKSI-4 and -5 fragments amplified from MP8E7 were quite different from each other and from the rest of the PKS sequences from this isolate. The amino acid sequence for PKSI-4 displayed 72% identity to the PKS from *Amycolatopsis orientalis* involved in the biosynthesis of the antibacterial compound ECO-0501 of a new chemical class [35]. The amino acid sequence for PKSI-5 displayed 96% identity to the PKS part of the NRPS-PKS fusion protein from *Streptomyces griseus* subsp. *griseus* NBRC 13350 (NC_010572.1)

The isolates MP6A2 and MP6D1 yielded 2 PKS sequences each. Both on the nucleotide and amino acid levels, MP6A2 PKSI-1 and MP6D1 PKSI-1 sequences were 100% identical to each other, and shared 83% identity with *S. coelicolor* cryptic PKS type I [36] The identity of the sequences suggests recent horizontal gene transfer between the two isolates, which has involved a PKS gene cluster. Two other PKS sequences amplified from these isolates, MP6A2 PKSI-2 and MP6D1 PKSI-2, encoded KS domains showing 94% and 96% identity, respectively, to the PKS and NRPS-PKS proteins encoded by two different gene clusters in *Streptomyces griseus* subsp. *griseus* NBRC 13350 [37]. Interestingly, the MPS06-A2 PKSI-2 sequence displayed 94% identity to the MP8E7 PKSI-5 sequence, also suggesting a relatively recent transfer of the corresponding gene between MP6A2 and MP8E7 isolates.

Isolates MP6A8, MP6C6, MP6C10 and MP9E12 each yielded one distinct KS domain encoding sequence. MP6A8 and MP6C6 PKSI sequences were 98% identical, suggesting recent horizontal gene transfer, and displayed 83% identity to the PKS type I from the cryptic gene cluster from *S. coelicolor*. The latter gene was different from the one showing closest match to the MP6A2/D1 PKS-1 sequences (see above), although apparently belonged to the same PKS cluster.

Both MP6C10 and MP9E12 PKS sequences were closely related (96% and 98% identity, respectively), to the NRPS-PKS fusion protein from *Streptomyces griseus* subsp. *griseus* NBRC 13350 [37]. They were also very similar to the PKS sequences MP6A2 PKSI-2 and MP8E7 PKSI-5, showing 93% and 96% identity, respectively.

**Figure 3.**
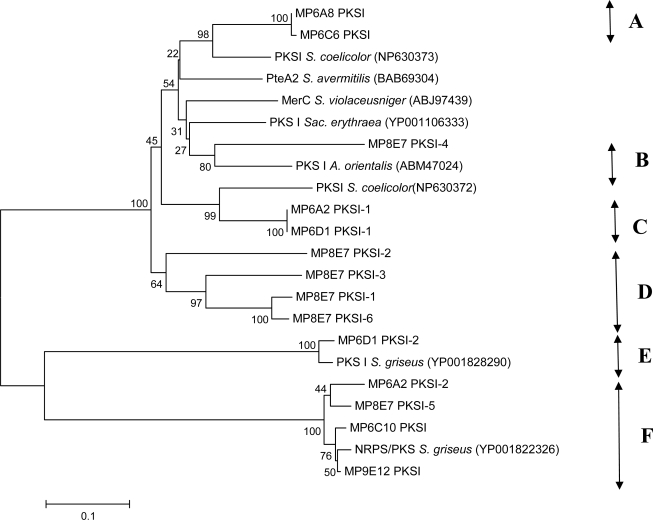
Phylogenetic relationship between PKS type I amino acid sequences from streptomycete isolates with identical partial 16S rDNA sequences. Closest matches from the BLAST searches are also included. Putative distinct KS domain types are indicated with letters (A, B, C etc). Numbers at tree nodes represent the number of times the topology to the right of the node was recovered in 1000 bootstrap re-samplings. Accession numbers for the sequences are given in parentheses. Scale bar represents the number of changes per amino acid position.

In order to visualize taxonomic relationship between the amino acid sequences encoded by the PCR-amplified fragments, a phylogenetic tree was constructed, which also included sequences from the best matches according to the BLAST search. The architecture of the tree, presented in Figure 3, clearly shows some discrepancy between the BLAST search and the phylogenetic analysis. For example, the PKSI-1,-2,-3 and -6 sequences from the isolate MP8E7 do not cluster with the corresponding best matches from the BLAST search, and form a separate branch on the tree. This suggests that the PKS gene cluster represented by these sequences might have evolved separately, and potentially can encode a novel polyketide metabolite.

Both BLAST and phylogenetic analyses suggest that we have been able to identify 6 distinct types of KS domains in 7 closely related (according to the 16S rDNA sequences) isolates. This does not necessarily mean that each type represents a distinct PKS gene cluster. In phylogenetic analysis, the KS domains are known to cluster not only according to their evolutionary relatedness, but also according to their substrate specificity [38]. Interestingly, one of the KS types belonging to the NRPS-PKS fusion protein (F) seems to be shared by at least 4 isolates and its coding DNA might have been subject to a relatively recent horizontal gene transfer. There is, however, no correlation between the presence of this KS type and antimicrobial activity profiles of the four isolates (Table 3). The latter suggests that the corresponding PKS cluster might be either not expressed in the conditions tested, or governs biosynthesis of a compound which is not detectable by the assays employed. The same might be true for the KS type C represented by the sequences MP6A2 PKSI-1 and MP6D1 PKSI-1, since the corresponding isolates have different inhibition patterns (see Table 3).

The analyses of the KS domains from selected isolates with identical 16S rDNA sequences do not enable solid conclusions about the nature of the compounds that may potentially be produced. However, the fact that unique KS types, such as B, D and E, seem to be isolate-specific, further supports the notion that streptomycete isolates can not be distinguished on the basis of 16S rDNA sequences alone. A more complex approach that includes PCR-based PKS and NRPS genome “scanning”, inoculation in a wide range of growth/production media, metabolite profiling and diverse biological assays with fractionated extracts is required to reveal the true potential for production of medically useful secondary metabolites.

## 3. Experimental Section

### 3.1. Sampling and isolation of Streptomycete bacteria from sea surface microlayer (Sampling sites and sample collection)

Samples were collected on the 22^nd^ of March 2004 at two sites (63°32,511 N, 010°48,797 E and 63º56,009 N, 010º91,020 E) in the Trondheim fjord, Norway. Steinvikholmen (sample 1) is a small islet situated approximately 200 m from the mainland, whereas the other sampling point was close to shore. The surface microlayer was collected using Teflon plates as earlier described [39]. The plates were immersed in water, gently lifted through the water surface, and the bacterioneuston scraped off using a rubber edge. Both samples were collected early in the morning during low tide, and 2 to 3 meters from the shoreline.

Samples were plated on selective agar plates (2% w/v), within 24 hours after collection, and was incubated at 20 ºC. Three different media was used; ½ ISP2; Malt extract (5 g), yeast extract (2 g), glucose (2 g), natural sea water (0.5 L) and distilled water (0.5 L), Kusters streptomycete isolation medium (modified); Glycerol (10 g), Casein (0.3 g), KNO_3_ (2 g), FeSO_4_*7 H_2_O (0.25 mg), H_2_SO_4_ (0.5 mg), natural sea water (0.5 L) and distilled water (0.5 L) and Actinomycete isolation medium without MgSO_4_ [40]. The pH of the isolation media was adjusted to pH 8.2. All media contained 50% sea water and was supplied with Cycloheximide (50 μl/ml) and Nalidixic acid (30 μl/ml). Selected isolates were transferred to ½ ISP2 agar to ensure pure colonies, and incubated for 16 days before storing as glycerol stock in micro well plates at −80 °C.

### 3.2. Extraction and antimicrobial assay

The selected strains were transferred to microwell filter plates (Nunc Silent screen nr 256073, Loprodyne 3.0 μm) with 80 μl of three different 1% agarose media (production media) to facilitate production of secondary metabolites. The production media (PM) were: PM2; Mannitol (20 g), soya bean flour (20 g), Clerol (antifoam, 0.5 g), dry yeast (3.4 g), agarose (10.0 g), tap water (1 L), PM3; Oatmeal (20 g), glycerol (2.5 g), FeSO_4_·7H_2_O (0.1 mg), MnCl_2_·4H_2_O (0.1 mg), ZnSO_4_·7H_2_O (0.01 mg), H_2_SO_4_ (0.1 mg), agarose (10 g), tap water (1 L), PM4; glucose (0.5 g), glycerol (2.5 g), oatmeal (5.0 g), soybean meal (5.0 g), yeast extract (1 g), casaminoacids (2.0 g), CaCO_3_ (1.0 g), Clerol (0.2 g), agarose (10 g) and tap water (1 L).

After an appropriate incubation time, 3 mm glass beads were added to the plates, and the strains were dried in the dark over night before extraction with 150 μl DMSO. The plates were shaken for 2 h at 1000 rpm before vacuum filtration (Event 4160, Eppendorf). These extracts were stored at −20 °C, and tested in agar diffusion assays for content of antagonistic compounds active against *Micrococcus luteus* (ATCC 9341)*, Candida albicans* (ATCC 10231) and *Escherichia coli* K12.

A variant of Burkholder agar diffusion assay [41] was used when screening for antimicrobial activity. Indicator agarose was prepared by mixing 1% agarose medium with 0.5-1% v/v indicator organism culture (OD_600_ = 3,6 _M. luteus_, 5,0 _C. albicans,_ 3,0 _E. coli_.), and poured into Petri dishes. LB agarose medium was used for *E. coli*, M19 for *C. albicans* and M1 for *M. luteus*. The media contained: M1; peptone (6.0 g), trypton (4.0 g), yeast extract (3.0 g), beef extract (1.5 g), dextrose (1.0 g), agarose (10 g) and tap water (1 L), pH 6,6. M19; beef extract (2.4 g), yeast extract (4.7 g), peptone (9.4 g), dextrose (10.0 g), NaCl (10.0 g), agarose (10 g) and tap water (1 L), pH 6,1.

DMSO-extracts were stamped manually from microwell plates onto the indicator agarose with the selected indicator organism. Approximately 2 μl of each extract was applied onto plates with 1.3 cm thick indicator agarose. The plates were preincubated for 3 to 4h at 4 °C, before incubating at 30 ºC over night. Extracts were defined as inhibiting if inhibition zones were ≥2mm larger than the diameter of the applied sample.

### 3.3 Cloning, sequencing and phylogenetic analysis

Based on morphology and inhibition patterns from the antimicrobial assays, subgrouping was performed, and candidates from each subgroup sequenced. PCR was performed directly on colonies or with isolated total-DNA as template. Total-DNA of the bacteria was isolated using DNeasy Blood & Tissue Kit (Qiagen) according to manufacturer’s protocol.

The primers BP_F27: 5’-AGA GTT TGA TCM TGG CTC AG-3’ and BP_R1492: 5’-TAC GGY TAC CTT GTT ACG ACT T-3’ [42], were used to amplify 1,5 kb of the 16S rRNA gene. The PCR was performed using initial denaturation at 94 °C for 4 minutes, followed by 35 cycles of 94 °C for 45 seconds, 55 °C for 20 seconds and 66 °C for 2 minutes. A final extension was performed at 70 °C for 5 minutes. PCR products were purified directly or after excision from agarose gel, using QIAquick Spin Kits according to the manufacturer’s instructions (Qiagen). Purified PCR-products were transformed into *E. coli* EZ competent cells after ligation into the pDrive cloning vector using the QIAGEN PCR-cloning Kit (Qiagen).

The 16S rRNA fragments were sequenced either from the pDrive-clones or directly after PCR. The primer M13 reverse: 5′-AACAGCTATGACCATG-3′ described in the Qiagen PCR Cloning Handbook (04/2001) was used for the pDrive-clones. Sequencing directly on the PCR products were performed with the same primers as for the PCR. The sequencing was performed using BigDye® Terminator v3.1 Cycle Sequencing Kit (Applied Biosystems). The sequencing program consisted of a initial step at 96 °C for 3 minutes, and 25 cycles of 96 °C for 30 seconds, 50 °C for 20 seconds and 60 °C for 4 minutes.

The phylogenetic analyses of the cloned sequences were performed using MEGA 4. A phylogenetic tree was constructed using neighbour-joining with 500 bootstrap replicates. Comparisons of the sequences with other available 16S rDNA sequences were done by BLAST searches to determine strain homology. The 16S rDNA sequence from *Micromonospora* sp DSM 44397 was included to root the tree.

### 3.4 PCR amplification of PKS and NRPS-genes

Bacterial modular type I PKS genes were amplified with the degenerate primers KSMA-F (5’-TS GCS ATG GAC CCS CAG CAG-3’) and KSMB-R (5’-CC SGT SCC GTG SGC CTC SAC-3’) [31]. PCR with these primers, amplifying the β-ketoacyl synthase (KS) domain (~700 bp), was performed using initial denaturation at 96 °C for 5 min, 35 cycles of 95 ºC for 1 min, 60 ºC for 1 min and 72ºC for 2 min. Final extension was performed at 72 ºC for 5 min.

For each reaction 200 μM dNTPs 20-40 ng total-DNA and 200 nM of each primer were used. Cloning of the fragments was performed as described for 16S rDNA Sequencing was performed by Eurofins MWG Operon.

### 3.5 Nucleotide sequence accession numbers

DNA sequences reported in this study have been deposited to GenBank under accession numbers FJ190540-FJ190569

## Figures and Tables

**Table 1.** Characteristics of the different isolate groups, when grown on ½ISP2 medium with 50% seawater for up to 14 days. SM = substrate mycelium, AM = aerial mycelium

**Group**	**Characteristics**		
	SM	AM	Other
**1**	Colourless	White	
**2**	Colourless	White	Produces yellow metabolite diffusing in solid media
**3**	Colourless	Greenish-white	
**4**	Colourless	Greenish-white	Produces yellow metabolite diffusing in solid media
**5**	Light brown	Light grey	
**6**	Brown /greenish	Grey	
**7**	Colourless /light brown	Light purple	
**8**	Red	None	
**9**	Red	White	
**10**	Yellow	None	Flaky

**Table 2.** Total number of streptomycete-like isolates from bacterioneuston, sample 1 and 2, grouped and sub-grouped according to antimicrobial activity and colony morphology. DMSO-extracts from all strains were tested for activity against *C. albicans* (C), *M. luteus* (M) and *E. coli* (E). Samples 1 and 2 contain 134 and 83 isolates, respectively S1 and S2 indicate sample 1 and sample 2, and G1-G10 indicate morphology groups 1- 10, (see Table 1). The percentages (S1 and S2 combined) of antifungal, antibacterial and no activity in each of the groups, G1-G10, are also given.

				**Group (G1-10) and sample number (S1, S2)**																				
**Inhibition**				**G1**		**G2**		**G3**		**G4**		**G5**		**G6**		**G7**		**G8**		**G9**		**G10**		
**Nr**	**C**	**M**	**E**	**S1**	**S2**	**S1**	**S2**	**S1**	**S2**	**S1**	**S2**	**S1**	**S2**	**S1**	**S2**	**S1**	**S2**	**S1**	**S2**	**S1**	**S2**	**S1**	**S2**	**Total**
**1**	**x**	**x**	**x**	6	7	0	1	5	0	2	0	1	0	0	0	0	0	1	4	0	2	0	0	29
**2**	**x**	**x**		6	1	0	0	0	0	0	0	0	0	1	0	0	0	0	0	0	0	0	0	8
**3**	**x**		**x**	9	4	0	0	0	0	0	0	1	0	0	0	0	0	0	0	0	0	0	0	14
**4**	**x**			28	14	4	0	0	2	0	0	4	3	3	3	0	0	0	1	0	0	0	0	62
**5**		**x**	**x**	4	7	1	3	2	2	7	2	1	0	0	1	0	0	2	8	3	1	1	0	45
**6**			**x**	5	2	0	0	0	1	0	0	0	0	0	0	2	0	0	0	0	0	1	0	11
**7**		**x**		2	0	0	0	0	0	4	0	1	1	0	0	0	0	0	0	0	0	0	0	8
**8**				12	7	2	0	1	0	0	0	5	4	7	2	0	0	0	0	0	0	0	0	40
Sum, no of isolates				72	42	7	4	8	5	13	2	13	8	11	6	2	0	3	13	3	3	2	0	217
Sum, no of isolates				114		11		13		15		21		17		2		16		6		2			
Antifungal (%)				66		45		54		13		43		41		0		38		33		0			
Antibacterial (%)				46		45		77		1 00		24		12		100		94		100		100			
No activity (%)				17		18		8		0		43		53		0		0		0		0			

**Table 3.** Names and inhibition patterns of isolates selected for PKS analysis. Activity is shown against *C. albicans* (C), *M. luteus* (M) and *E. coli* (E). Morphology group and sample number are indicated.

**Isolate**	**Morphology group**	**Sample number**	**Inhibition**		
			**C**	**M**	**E**
MP6A2	G4	S1		**x**	**x**
MP6A8	G3	S1	**x**	**x**	**x**
MP6C6	G4	S1		**x**	
MP6C10	G1	S1	**x**	**x**	
MP6D1	G2	S1	**x**		
MP8E7	G10	S1			**x**
MP9E12	G1	S2			
